# Visual Integration Mechanisms in Young Adults with High and Low Autistic Traits

**DOI:** 10.3390/life16091475

**Published:** 2026-09-03

**Authors:** Sara Bertoni, Sandro Franceschini, Simona Carbone, Chiara Filotto, Martina Mancarella, Giovanna Puccio, Andrea Pavan, Simone Gori, Andrea Facoetti

**Affiliations:** 1 Developmental Cognitive Neuroscience Lab, Department of General Psychology, University of Padova, 35131 Padova, Italy; 2 Child and Adolescent Neuropsychiatry Unit, Department of Medicine and Technological Innovation, University of Insubria, 21100 Varese, Italy; 3 Department Psychology, University of Bologna, 40127 Bologna, Italy; 4 Department of Human and Social Sciences, University of Bergamo, 24129 Bergamo, Italy

**Keywords:** autism trait continuum, self-reported social traits, visual integration, visual attention, global perception, object perception

## Abstract

**Background:** Social interaction requires the efficient extraction and integration of visual information. Although atypicalities in global processing and visual perception have been reported in autism, their relationship with self-reported social-skills-related autistic traits remains unclear. **Methods:** Using an extreme-groups design, 179 university students completed the Autism-Spectrum Quotient (AQ). Individuals with scores at least ±1 SD were selected to form high-AQ (*n* = 20) and low-AQ (*n* = 22) groups. Participants completed two complementary psychophysical tasks assessing object perception under increasing visual noise and global–local visual processing. **Results:** The high-AQ group showed reduced recognition under high visual noise and slower responses under the global-incongruent condition. In the exploratory regression, visual-noise accuracy explained significant variance when entered alone, whereas global-incongruent Navon performance explained incremental variance and was the only significant unique contributor in the final model (R^2^ = 0.280). **Conclusions:** These findings suggest that, within the selected extreme-AQ sample, AQ Social Skills scores are associated with complementary visual mechanisms responsible for extracting relevant information from noisy environments and integrating spatially distributed visual information into coherent global representations. These behavioural findings are consistent with a possible link between visual processing and self-reported social traits and with theoretical models proposing a role for magnocellular-dependent dorsal visual computations in social perception, although these neural mechanisms were not directly assessed.

## 1. Introduction

Autistic traits are increasingly recognised as continuously distributed across the general population, with autism spectrum disorder (ASD) representing the upper end of a quantitative continuum rather than a categorically distinct condition [1]. This dimensional perspective is consistent with contemporary transdiagnostic frameworks, including the Research Domain Criteria (RDoC) [2], which emphasize continuous variation in neurocognitive mechanisms across clinical and non-clinical populations. Investigating autistic traits in typically developing individuals therefore provides an opportunity to identify candidate neurocognitive mechanisms underlying social functioning before they reach clinical levels [3].

Although ASD is primarily defined by persistent differences in social communication and interaction [4], converging evidence indicates that atypical visual perception constitutes a core feature of the autistic phenotype rather than a secondary consequence of social impairment [5,6]. We propose that a common denominator of these perceptual differences may be reduced efficiency in integrating spatially distributed visual information, a computational process that is fundamental for object perception, scene perception, and social interaction. Individuals with ASD or infants at-risk show differences in the perception of motion, biological motion, visual attention disengagement and deployment, contextual processing and object perception, suggesting that early perceptual mechanisms may contribute to higher-order socio-communicative functioning [7,8,9,10,11].

One of the most extensively investigated perceptual characteristics of ASD concerns the balance between local and global visual processing [12]. According to the Weak Central Coherence account, autistic individuals preferentially process local details at the expense of global integration [13,14]. Alternatively, the Enhanced Perceptual Functioning model proposes that superior local processing reflects enhanced low-level perceptual mechanisms rather than impaired global processing [15]. Although these accounts differ in their interpretation, both suggest that global visual integration represents a key computational challenge in ASD. Meta-analytic evidence further indicates that global processing is often less efficient rather than absent, particularly when tasks require integrating multiple visual elements over time or under perceptually demanding conditions [9].

Recent developments in visual neuroscience suggest that these findings may be better understood within a broader attentional and perceptual framework [10]. Rather than reflecting a deficit in global perception per se, successful global processing depends on the ability to flexibly expand the attentional window (“attentional zoom-out”) to integrate spatially distributed information into coherent perceptual representations [16,17]. Previous work has consistently shown that children with ASD exhibit reduced efficiency in attentional zoom-out mechanisms [18,19,20], affecting coherent motion perception, global attentional deployment, surround suppression, and socio-communication behaviours [18,21,22,23]. These findings are consistent with the established role of the right-lateralised dorsal frontoparietal attention network in attentional disengagement and spatial reorienting [24,25]. In line with this framework, transient disruption of the right frontal eye fields by transcranial magnetic stimulation selectively induces an inflexible attentional focus, directly supporting a causal role of this network in attentional zooming-out [26]. Importantly, similar attentional mechanisms can already be measured during infancy [27] and reduced attentional disengagement or reorienting during the first year of life predicts later socio-communicative functioning and ASD outcomes [8,11,28,29]. Together, these findings suggest that attentional zoom-out represents an early neurocognitive mechanism linking visual perception to social development.

This attentional account is also consistent with emerging neurobiological evidence of social perception [10]. Traditionally, visual perception has been explained by the interaction between the ventral “what” pathway and the dorsal “where” pathway [30]. However, growing evidence indicates that the dorsal visual stream contributes directly to perceptual computations by extracting global structural descriptions of objects that subsequently facilitate ventral object recognition [31,32]. More recently, Pitcher and Ungerleider [10] proposed the existence of a third visual pathway dedicated to social perception—mainly lateralised in the right lateral cortex—projecting from early visual cortex through the motion-selective area MT/V5 toward the superior temporal sulcus, where dynamic social signals, such as eye gaze, facial expressions and biological motion, are processed [33,34,35].

Interestingly, recent ultra-high-field (7 Tesla) functional MRI has revealed selective functional alterations within the magnocellular subdivision of the lateral geniculate nucleus in adults with ASD, providing direct evidence that atypical visual processing may originate at very early stages of the visual system [36]. Together with behavioural evidence demonstrating atypical attentional selection and perceptual integration in ASD, these findings raise the possibility that social difficulties may partly emerge from inefficient extraction and integration of behaviourally relevant visual information across complex scenes.

One powerful approach to investigate this hypothesis is to examine visual perception under conditions of external noise. Visual-noise paradigms provide a well-established method for quantifying the efficiency with which the visual system extracts behaviourally relevant information from competing sensory input [37]. Importantly, successful perception under noisy conditions depends on efficient attentional selection mechanisms that enhance relevant signals while suppressing irrelevant visual information [38], processes that appear atypical in ASD [6]. Because real-world social environments are inherently cluttered and visually complex, efficient social perception likewise depends on extracting behaviourally relevant visual information while ignoring competing irrelevant input [39]. Likewise, successful performance on global Navon [40] stimuli require expanding attention beyond local elements to encode the global configuration [9,41]. Although object perception in noise and global perceptual processing have independently been implicated in ASD [5,37], little is known about whether these perceptual functions are associated with AQ Social Skills subscale scores.

The present study addressed this question using an extreme-groups design in young adults with high and low autistic traits. Participants completed the following two complementary psychophysical paradigms: an isoluminant object-recognition task under increasing levels of visual noise, probing the extraction of behaviourally relevant visual signals under degraded conditions, and a global–local Navon task assessing global perceptual integration, which requires the flexible expansion of the attentional focus (attentional zoom-out). We hypothesised that individuals with higher autistic traits would show reduced performance whenever successful perception depended on integrating spatially distributed visual information, particularly under high levels of external visual noise and during the global-incongruent Navon condition, in which the interference from local elements must be overcome to correctly identify the global configuration. Finally, we examined whether the two behavioural measures were associated with AQ Social Skills subscale scores within the selected extreme-AQ sample. We predicted that object perception under visual noise and global visual integration would make complementary contributions to variation in AQ Social Skills subscale scores within the selected extreme-AQ sample, consistent with the hypothesis that self-reported social-skills-related autistic traits are associated with attentional mechanisms supporting the efficient extraction and integration of behaviourally relevant visual information across complex scenes.

## 2. Materials and Methods

### 2.1. Participants

Participants were recruited at the University of Padua using voluntary convenience sampling through different bachelor’s and master’s degree programmes in psychology. The initial screening was open to students enrolled in these programmes who wished to participate. No financial compensation, course credit, or additional examination points were provided. All participants were native Italian speakers and reported normal or corrected-to-normal vision. No additional exclusion criteria were applied.

A total of 179 university students completed the Autism-Spectrum Quotient (AQ) [1]. The AQ is a 50-item self-report questionnaire designed to quantify autistic traits in adults. It comprises five 10-item subscales assessing Social Skills, Attention Switching, Attention to Detail, Communication, and Imagination. The original validation demonstrated good discriminative validity and test–retest reliability, with a Cronbach’s alpha of 0.77 for the Social Skills subscale [1]. The Italian version was shown to be equivalent to the original English version and demonstrated internal consistency coefficients of 0.76 for the total score and 0.68 for the Social Skills subscale [42]. The mean AQ total score in the overall sample was 16.95 (SD = 6.19). The AQ was selected because autistic traits are considered to be continuously distributed throughout the general population, with ASD representing the upper end of this continuum. To investigate visual integration mechanisms as a function of autistic trait expression within a non-clinical population, an extreme-groups approach was adopted (e.g., [11]). Participants scoring at least one standard deviation above the sample mean were classified as having high autistic traits (high-AQ), whereas participants scoring at least one standard deviation below the sample mean were classified as having low autistic traits (low-AQ). This selection procedure resulted in 42 participants entering the psychophysical phase: 20 in the high-AQ group and 22 in the low-AQ group. All 42 participants meeting the predefined cut-offs accepted the invitation to the psychophysical phase; none declined participation or subsequently withdrew from the study. The experimental battery was designed to investigate the following two complementary components of visual integration: (i) the extraction of behaviourally relevant visual information under increasing levels of external noise and (ii) the integration of spatially distributed visual information into coherent global representations.

The groups did not differ significantly in age, t_(40)_ = 0.75, *p* = 0.459 (high-AQ: M = 20.95 years, SD = 2.89; low-AQ: M = 20.36 years, SD = 2.17), or sex distribution (high-AQ: 3 males and 17 females; low-AQ: 3 males and 19 females; χ^2^(1, N = 42) = 0.016, *p* = 0.900; Fisher’s exact test, *p* = 0.999).

Although sex distribution did not differ between the groups, the predominantly female composition of the sample should be considered when interpreting the findings and limits their generalisability across sexes.

Because data availability differed across tasks, the analytical sample size was reported separately for each analysis. The visual-noise analysis included 41 participants (20 high-AQ and 21 low-AQ), because the corresponding computerised task data were unavailable for 1 participant in the low-AQ group. The Navon analysis included 38 participants (20 high-AQ and 18 low-AQ), because 4 participants in the low-AQ group did not complete the paper-and-pencil Navon task. The regression analysis included the 37 participants (20 high-AQ and 17 low-AQ) with complete AQ Social Skills scores and valid data for both visual measures. Analyses were conducted using complete cases, with listwise exclusion within each analysis, and no missing values were imputed.

### 2.2. Psychophysical Tasks

#### 2.2.1. Visual Signal Extraction Under External Noise

To assess the ability to extract behaviourally relevant visual information under degraded viewing conditions, participants completed a static isoluminant grating orientation task adapted from previous studies [43,44,45]. The paradigm systematically manipulates the amount of external visual noise superimposed on the target stimulus, requiring participants to segregate the relevant visual signal from noisy information. Successful performance, therefore, depends on efficiently extracting and integrating the target configuration despite progressively increasing perceptual degradation.

Stimuli consisted of high-spatial-frequency coloured isoluminant gratings presented at one of four possible orientations and embedded within five levels of coloured random isoluminant visual noise. The task has previously been used to investigate visual signal extraction under noisy conditions [43,44,45].

The task was administered using E-Prime 2.0 (Psychology Software Tools, Inc., Pittsburgh, PA, USA). Stimuli were presented centrally on a computer monitor in a dimly lit (1.5 cd/m^2^) quiet room. Participants were seated at a viewing distance of 40 cm. Each trial began with a central fixation cross (0.5°). After 500 ms, a circular isoluminant grating (7.4°, 40 cd/m^2^; spatial frequency of 1.4 cycles/degree) was presented for 51 ms. The grating appeared at one of four orientations (40°, 85°, 130°, or 175°; chance level = 0.25). Five levels of coloured random visual noise were superimposed to the stimulus ranging from 0 (absence of noise) to 4 (maximum level of noise).

**Figure 1:**
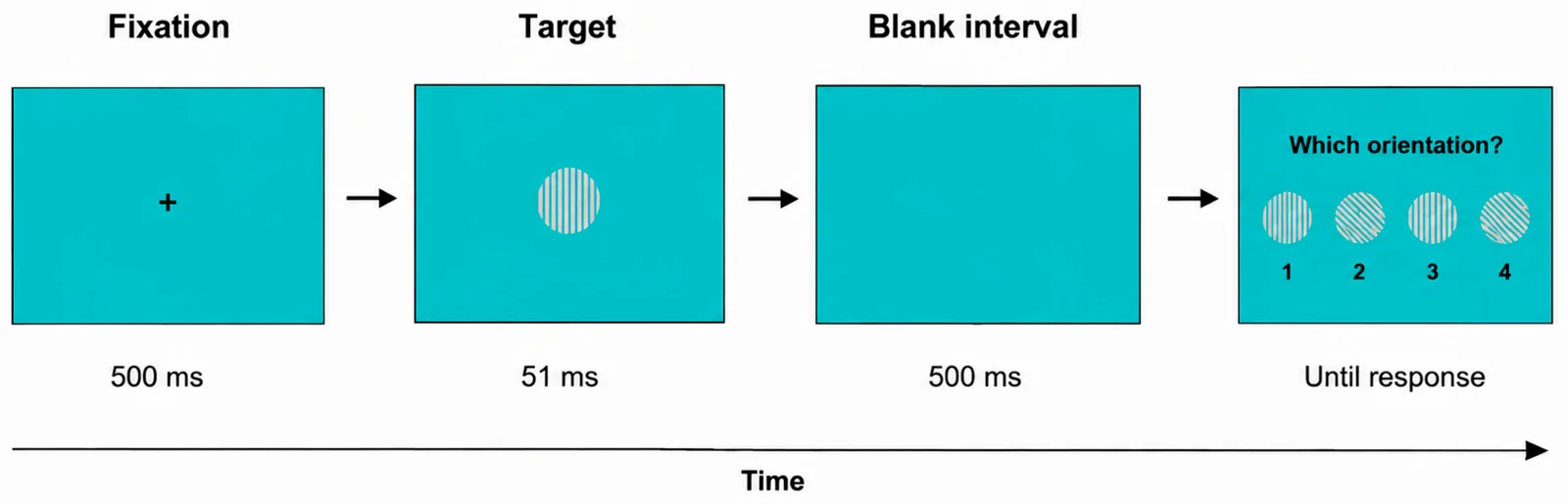
Static isoluminant grating orientation task with 5 levels of superimposed random noise.

Participants identified the grating orientation by selecting one of four response alternatives displayed on the screen. The task was accuracy-based: the response accuracy was recorded, whereas the response times were not measured. The task consisted of 40 trials (4 orientations × 5 noise levels × 2 repetitions). The visual noise level served as the within-subject factor and the response accuracy as the dependent measure (Figure 1).

#### 2.2.2. Global Visual Integration

Global and local visual integration was assessed using a paper-and-pencil Navon task adapted from Franceschini et al. [41]. Whereas the local condition primarily required focusing attention on individual constituent elements (attentional zoom-in), the global condition required expanding the attentional focus (attentional zoom-out) in order to integrate spatially distributed local elements into a coherent global representation.

Participants completed both the global and local versions of the task, which were administered in a counterbalanced order across participants. During the global condition, they were instructed to identify the large (global) figure while ignoring its constituent local elements. During the local condition, they identified the smaller composing figures while ignoring the global configuration.

**Figure 2:**
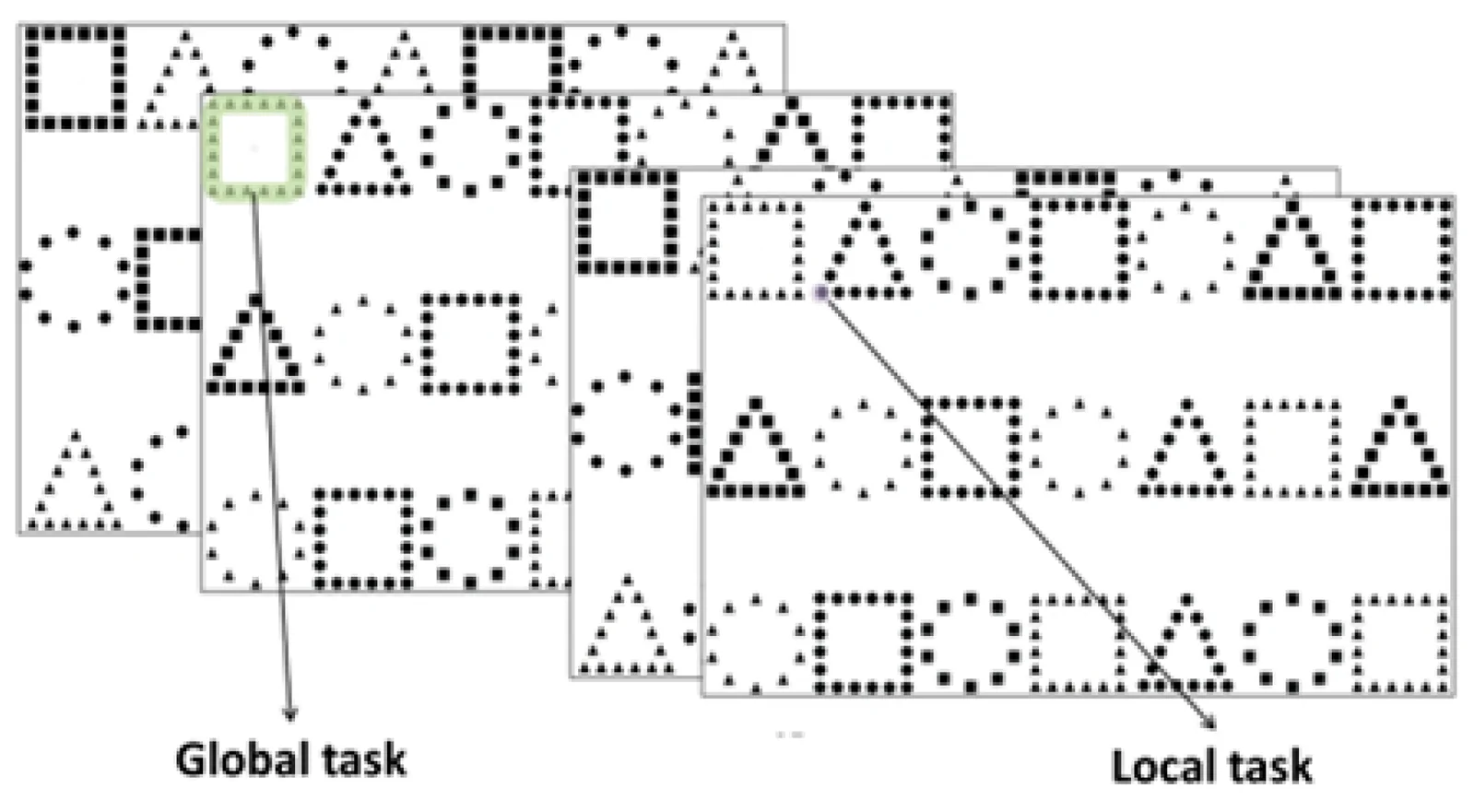
The global–local Navon task [41].

Stimuli were either congruent (e.g., a large square composed of small squares) or incongruent (e.g., a large square composed of small triangles), allowing for manipulation of the interference generated by conflicting local and global information (Figure 2).

## 3. Statistical Analysis

Statistical analyses were performed using IBM SPSS Statistics, version 32.0 (IBM Corp., Armonk, NY, USA). Analyses were conducted using complete cases for the variables included in each model. The visual-noise analysis included 41 participants, the Navon analysis included 38 participants, and the regression analysis included 37 participants. Missing values were excluded listwise within each analysis and were not imputed. Statistical significance was defined as *p* < 0.05. Unless otherwise specified, tests were two-tailed. Effect sizes for the analyses of variance were reported as partial eta squared.

For both mixed-design ANOVAs, condition-specific distributions and potential extreme observations were examined using Shapiro–Wilk tests, Q–Q plots, and boxplots. Homogeneity of variance was assessed using Levene’s tests, and equality of the covariance matrices was evaluated using Box’s test.

### 3.1. Visual Signal Extraction Under External Noise

Accuracy in the visual signal-extraction task was analysed using a mixed-design ANOVA, with Visual Noise (Levels 0–4) as the within-participant factor and Group (high-AQ vs. low-AQ) as the between-participant factor. Sphericity was assessed using Mauchly’s test, and Greenhouse–Geisser-corrected degrees of freedom and *p*-values were reported when this assumption was violated.

Because the hypothesis specifically predicted that group differences would increase as visual noise increased, the primary test was a planned linear contrast for the Visual Noise × Group interaction. Between-group comparisons at the two highest noise levels (Levels 3 and 4) constituted a hypothesis-driven family of planned directional contrasts, whereas comparisons at the three lower levels constituted a separate family of exploratory contrasts. Because the directional hypothesis predicted lower accuracy in the high-AQ group, these simple between-group comparisons were evaluated using one-tailed tests. Within each family, *p*-values were adjusted using the Holm procedure.

### 3.2. Global Visual Integration

Response times in the Navon task were analysed using a mixed-design ANOVA, with Task (global vs. local) and Condition (congruent vs. incongruent) as within-participant factors and Group as the between-participant factor. Because both within-participant factors had only two levels, sphericity was satisfied by definition. Accuracy was not submitted to inferential analysis because error rates were at floor level across all conditions.

The between-group comparison in the global-incongruent condition represented the single planned contrast derived from the hypothesis stated in the Introduction. Because Levene’s test indicated unequal variances in this condition, the comparison was evaluated using Welch’s unequal-variances *t* test. As a single hypothesis-driven contrast, it was not adjusted for multiple comparisons.

The remaining simple comparisons were used to decompose the Task × Condition × Group interaction and were considered exploratory follow-up analyses. Holm corrections were applied separately to the following three conceptually defined families: the three remaining between-group comparisons, the four congruency comparisons across Group × Task combinations, and the two global–local comparisons on incongruent condition. Directional congruency comparisons, reflecting the prediction of slower performance in incongruent than congruent conditions, were evaluated using one-tailed tests. All other Navon comparisons were two-tailed. Adjusted *p*-values are explicitly identified in the Results (Section 4).

### 3.3. Exploratory Regression Analysis

As a secondary exploratory analysis, a hierarchical multiple regression was conducted using the standardised AQ Social Skills subscale score as the dependent variable. Accuracy at the highest level of external visual noise was entered at Step 1, followed by response time in the global-incongruent Navon condition at Step 2. The incremental contribution of the second predictor was evaluated using the change in R^2^ between the two steps.

Linearity and homoscedasticity were evaluated using plots of standardised residuals against predicted values. Residual normality was assessed using histograms, normal probability plots, and the Shapiro–Wilk test. Multicollinearity was assessed using tolerance and variance inflation factor values. Potentially influential observations were examined using studentised deleted residuals, Mahalanobis distance, leverage values, and Cook’s distance. Because the residuals departed significantly from normality, inference for the regression coefficients was supplemented with bias-corrected and accelerated 95% bootstrap confidence intervals and two-tailed bootstrap *p*-values based on 5000 resamples.

Because participants were selected on the basis of extreme AQ total scores, of which the Social Skills subscale is a component, the regression was interpreted as an exploratory analysis within the selected sample and not as an estimate of associations across the full distribution of autistic traits.

#### Sample-Size Determination and Statistical Sensitivity

No formal a priori power analysis was conducted because the psychophysical sample size was determined by applying the predefined extreme-group cut-offs to the initial AQ screening sample. Sensitivity analyses were subsequently conducted using G*Power, version 3.1.9.7 (Heinrich Heine University Düsseldorf, Düsseldorf, Germany), with the power set at 0.80 and the alpha set at 0.05, to quantify the minimum effect sizes detectable in the final analytical samples.

For the Visual Noise × Group interaction (N = 41), the minimum detectable effect was f = 0.260, corresponding to a partial eta squared of 0.063. This calculation assumed an average correlation of r = 0.134 among the five repeated measures and a Greenhouse–Geisser epsilon of 0.688. For the Task × Condition × Group interaction in the Navon task (20 high-AQ and 18 low-AQ participants), the minimum detectable standardised between-group difference in the within-participant Task × Condition contrast was d = 0.935. For the regression analysis (N = 37, two predictors), the minimum detectable overall effect was f^2^ = 0.285, corresponding to approximately R^2^ = 0.222. The minimum detectable incremental effect of one additional predictor in the final two-predictor model was f^2^ = 0.225.

## 4. Results

### 4.1. Visual Signal Extraction Under External Noise

Preliminary diagnostic analyses indicated no inequality of the covariance matrices, Box’s M = 11.77, *p* = 0.811, and no violation of homogeneity of variance at any noise level (all Levene’s ps ≥ 0.114). Departures from normality at Noise Levels 0 and 1 and, to a lesser extent, at Level 2 reflected ceiling effects and the discrete nature of the accuracy scores. No observations exceeded approximately three standard deviations from the corresponding group mean. Distributions at Levels 3 and 4 did not depart significantly from normality (all Shapiro–Wilk ps ≥ 0.132). Mauchly’s test indicated a violation of sphericity, W = 0.259, χ^2^(9) = 50.55, *p* < 0.001; Greenhouse–Geisser-corrected results are, therefore, reported for effects involving Visual Noise (epsilon = 0.688).

As expected, increasing external visual noise strongly reduced recognition accuracy, yielding a significant main effect of Visual Noise, F_(2.75,107.35)_ = 316.48, *p* < 0.001, η^2^_p_ = 0.89. A significant main effect of the Group was also observed, F_(1,39)_ = 6.82, *p* = 0.013, η^2^_p_ = 0.149, indicating overall lower performance in participants with higher autistic traits.

**Figure 3:**
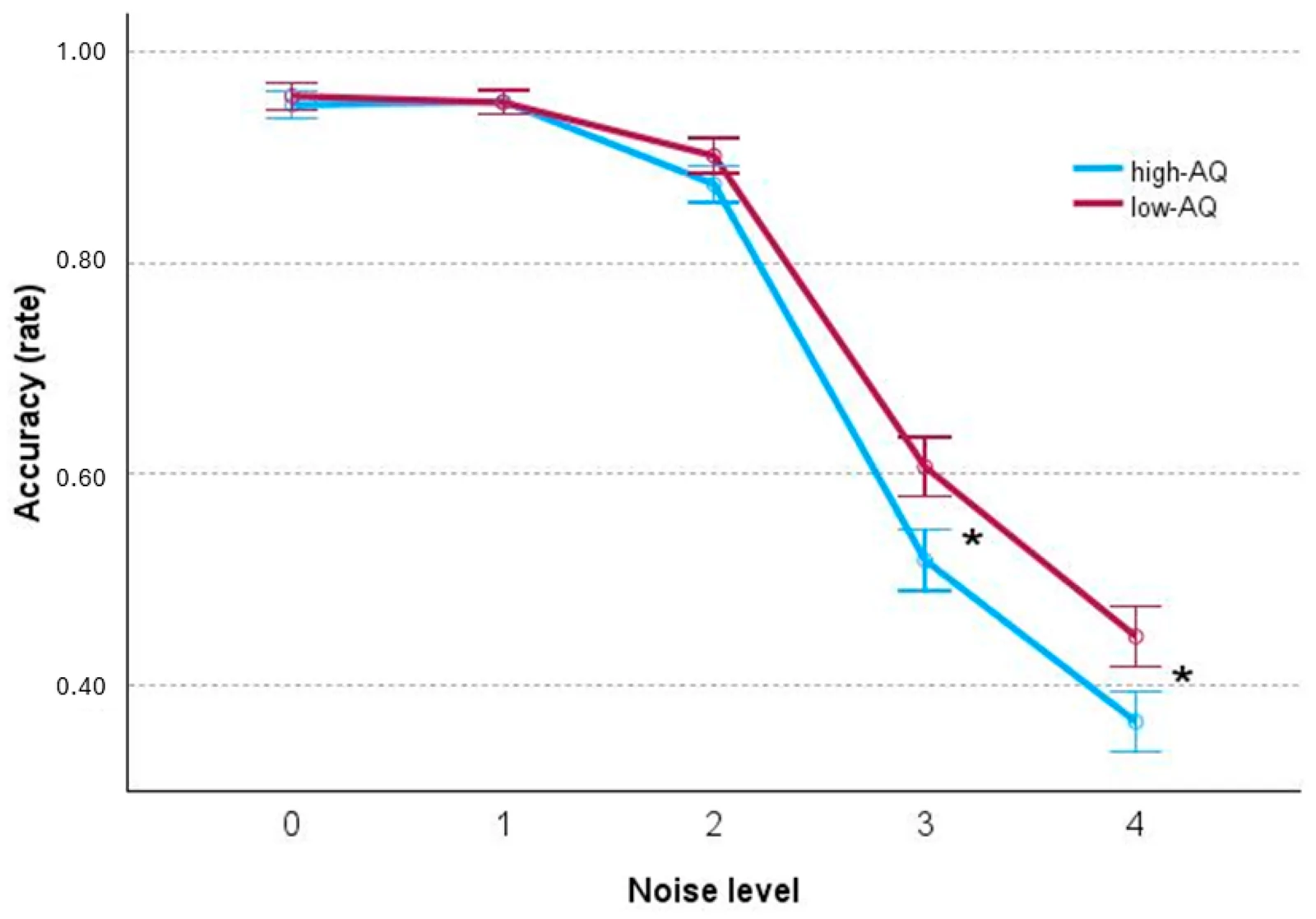
Performance in the visual signal-extraction task across five levels of external visual noise in participants with high-AQ and low-AQ traits. Error bars represent ±1 SEM. Asterisks indicate significant differences.

The Greenhouse–Geisser-corrected omnibus Visual Noise × Group interaction was not statistically significant, F_(2.75,107.35)_ = 2.12, *p* = 0.107, η^2^_p_ = 0.052. However, the planned linear component of the Visual Noise × Group interaction was significant, F_(1,39)_ = 4.40, *p* = 0.042, η^2^_p_ = 0.101, consistent with the predicted increase in the group difference as external visual noise increased (Figure 3).

Planned between-group comparisons indicated no significant differences at Noise Levels 0–2 (all Holm-adjusted ps ≥ 0.408). In contrast, the high-AQ group showed significantly lower accuracy than the low-AQ group at Level 3 (M = 0.519 vs. 0.607, Holm-adjusted *p* = 0.035) and Level 4 (M = 0.366 vs. 0.446, Holm-adjusted *p* = 0.035).

Taken together, the planned linear and level-specific contrasts indicated that group differences were most evident when successful perception required extracting behaviourally relevant information from highly degraded visual input.

### 4.2. Global Visual Integration

Examination of the condition-specific distributions indicated a departure from normality only for the global-congruent condition in the low-AQ group, W = 0.878, *p* = 0.024; all remaining distributions did not depart significantly from normality (all Shapiro–Wilk ps ≥ 0.055). No observations exceeded approximately three standard deviations from the corresponding group mean. Box’s test did not indicate inequality of the covariance matrices, Box’s M = 18.50, F = 1.63, *p* = 0.093. Levene’s tests were non-significant for three of the four conditions (all ps ≥ 0.058), but indicated unequal variances in the global-incongruent condition, F(1,36) = 4.82, *p* = 0.035. The planned comparison for this condition was, therefore, evaluated using Welch’s *t* test.

The analysis revealed significant main effects of Task, F_(1,36)_ = 8.75, *p* = 0.005, η^2^_p_ = 0.20, indicating a typical global processing advantage, as participants responded faster in the global than in the local task (M = 14.87 vs. 15.64, M difference = −0.77, *p* = 0.005). In addition, there was a significant main effect of Condition, F_(1,36)_ = 6.47, *p* = 0.015, η^2^_p_ = 0.15, reflecting typical faster responses in the congruent than incongruent conditions (M = 14.90 vs. 15.61, M difference = −0.71, *p* = 0.015).

**Figure 4:**
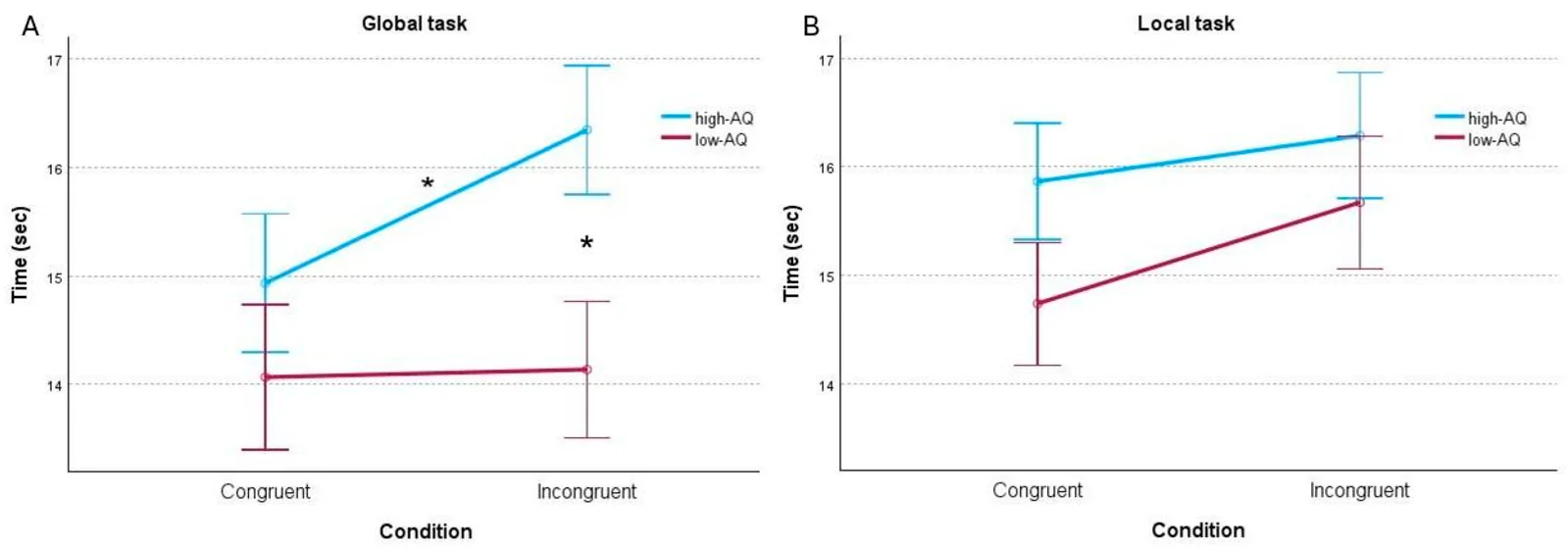
Performance in the global (**A**) and local (**B**) Navon tasks as a function of congruency in participants with high-AQ and low-AQ traits. Error bars represent ±1 SEM. Asterisks indicate significant differences.

Crucially, a significant Task × Condition × Group interaction emerged, F_(1,36)_ = 4.57, *p* = 0.039, η^2^_p_ = 0.11 (Figure 4). The planned comparison showed that the high-AQ group required longer response times in the global-incongruent condition (M = 16.34) than the low-AQ group (M = 14.14), Welch’s t (29.16) = 2.64, *p* = 0.013, 95% CI for the mean difference [0.50, 3.91]. The remaining between-group comparisons were non-significant after Holm correction (all Holm-adjusted ps ≥ 0.471).

Holm-adjusted within-group comparisons further clarified the three-way interaction. The high-AQ group showed a significant congruency effect in the global task (M difference = −1.41, Holm-adjusted *p* = 0.004), but not in the local task (M difference = −0.42, Holm-adjusted *p* = 0.436). In the low-AQ group, the congruency effects were not significant in either the global task (M difference = −0.07, Holm-adjusted *p* = 0.439) or the local task (M difference = −0.93, Holm-adjusted *p* = 0.162).

Finally, under the incongruent condition, the low-AQ group responded significantly faster in the global than in the local task (M = 14.14 vs. 15.67, M difference = −1.53, Holm-adjusted *p* = 0.003), whereas no global–local difference emerged in the high-AQ group (M = 16.34 vs. 16.29, M difference = 0.06, Holm-adjusted *p* = 0.898).

Within the selected sample, group differences were, therefore, most evident when global processing required integrating spatially distributed information while overcoming interference from competing local elements.

### 4.3. Exploratory Regression Analysis

As a secondary exploratory analysis, a hierarchical multiple regression was conducted in the 37 participants with complete data, using the standardised AQ Social Skills subscale score as the dependent variable. Because the sample had been selected using extreme AQ total scores, of which the Social Skills subscale is a component, the analysis was interpreted as describing associations within the selected sample rather than across the full autism-trait continuum.

Diagnostic analyses indicated no pronounced violations of linearity or homoscedasticity and negligible multicollinearity (tolerance = 0.930; VIF = 1.075). No strongly influential observations were identified (maximum absolute studentised deleted residual = 2.216; maximum Mahalanobis distance = 10.840; maximum Cook’s distance = 0.101). One observation showed elevated leverage (0.301) but was retained because it was not associated with an extreme residual or strong overall influence. Residuals departed significantly from normality, W = 0.898, *p* = 0.003; therefore, coefficient estimates were supplemented with bias-corrected and accelerated bootstrap inference based on 5000 resamples.

**Figure 5:**
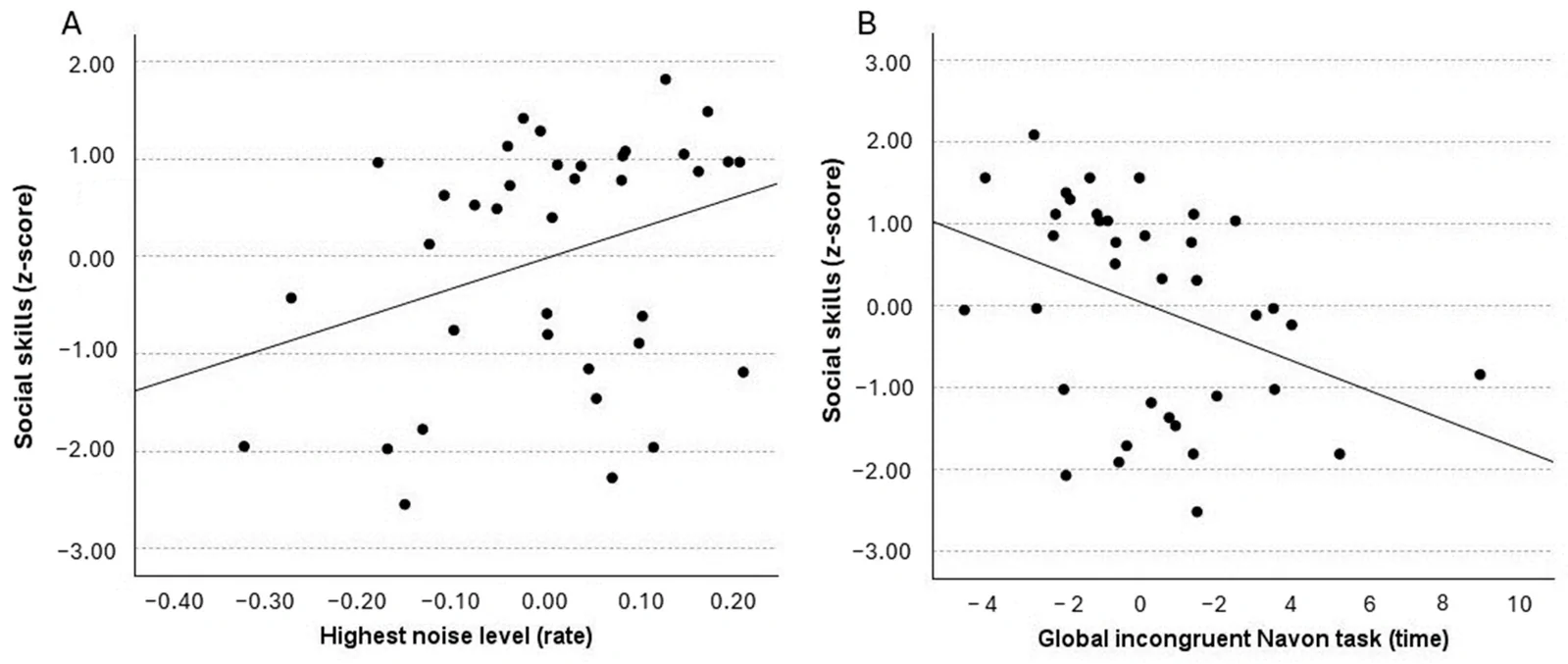
Associations of AQ Social Skills scores with visual signal extraction (**A**) and global visual integration (**B**).

In the first step, performance under the highest external-noise condition of the visual signal-extraction task was entered as the predictor. This model was significant, accounting for 15.8% of the variance in AQ Social Skills, R^2^ = 0.158, F_(1,35)_ = 6.56, *p* = 0.015 (Figure 5A). Visual-noise accuracy was a significant predictor at this step, B = 4.205, standardised β = 0.397, bootstrap *p* = 0.003, 95% BCa CI [1.213, 6.799].

In the second step, time under the global-incongruent Navon condition was entered as an additional predictor. This significantly improved the model fit, explaining an additional 12.2% of the variance, ΔR^2^ = 0.122, F-change _(1,34)_ = 5.75, *p* = 0.022 (Figure 5B). The final model explained 28% of the variance in the AQ Social Skills scores, R^2^ = 0.280, F_(2,34)_ = 6.60, *p* = 0.004.

In the final model, global-incongruent Navon performance made a significant unique contribution, B = −0.176, standardised β = −0.362, t = −2.40, *p* = 0.022; bootstrap *p* = 0.009, 95% BCa CI [−0.318, −0.081]. By contrast, the unique contribution of visual-noise accuracy did not reach the conventional significance threshold, B = 3.191, standardised β = 0.301, t = 2.00, *p* = 0.054; bootstrap *p* = 0.051, 95% BCa CI [−0.212, 6.172].

Thus, within this selected sample, visual-noise accuracy explained significant variance when entered alone, whereas global-incongruent Navon performance explained significant incremental variance and was the only measure to make a statistically significant unique contribution in the final model. Given the extreme-groups design, these exploratory findings should not be interpreted as estimates of associations across the full autism-trait continuum or as evidence of a direct relationship with real-world social functioning.

## 5. Discussion

**Figure 6:**
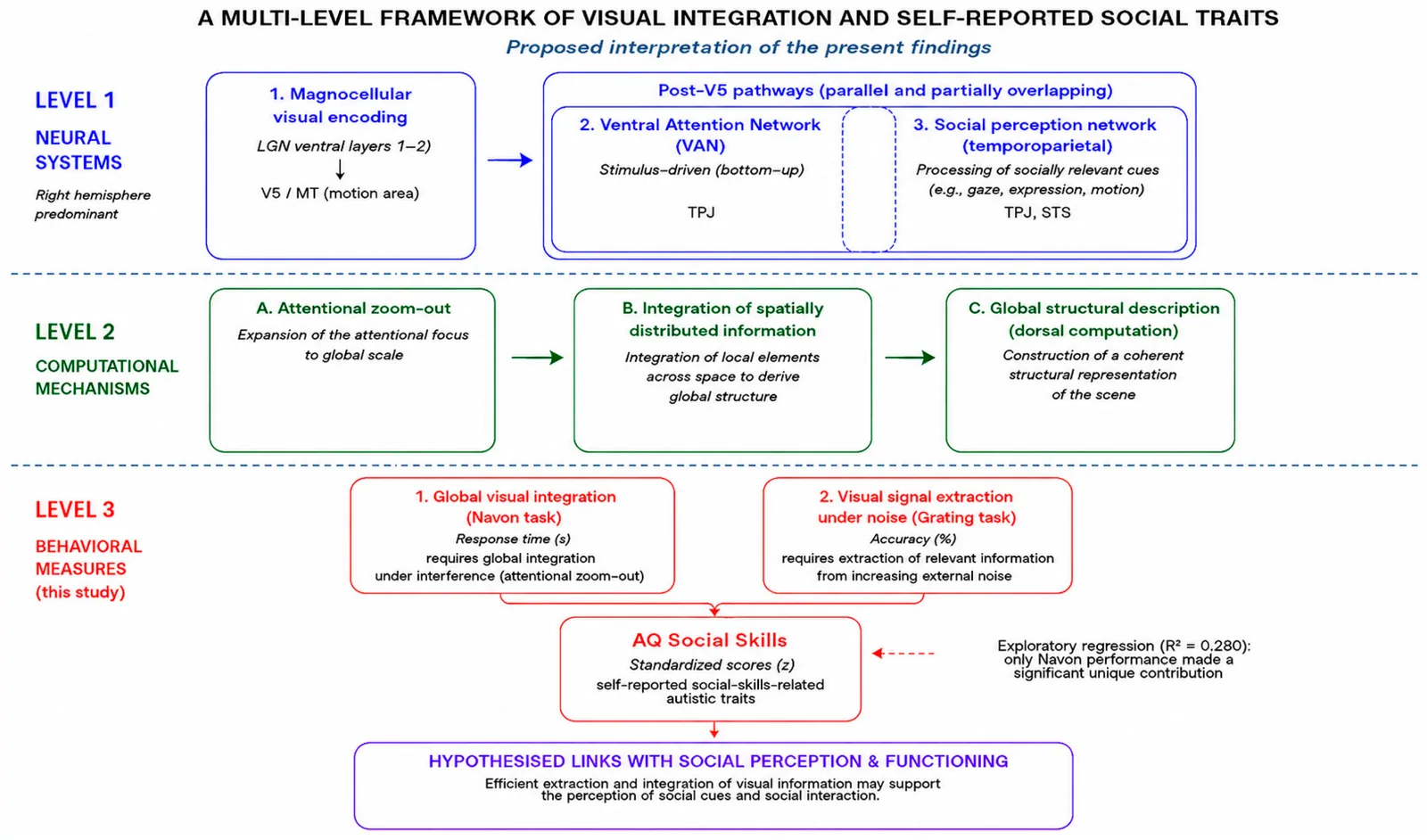
Proposed theoretical framework relating visual integration mechanisms to self-reported social-skills-related autistic traits. Early magnocellular visual encoding is hypothesised to feed two partially overlapping right-hemisphere pathways—the ventral attention network and the social perception network—which may support the integration of spatially distributed visual information and the construction of global structural representations. The present study assessed only behavioural indices of visual signal extraction under external noise (grating task) and global visual integration (Navon task), rather than the proposed neural pathways. This framework is a theoretical interpretation integrating previous evidence with the present behavioural findings and does not represent a direct demonstration of the underlying neural circuitry.

The present study compared two aspects of visual processing in young adults selected for high or low AQ scores. The framework presented in Figure 6 is a hypothesis-generating interpretation integrating the present behavioural results with prior literature rather than a model derived from neurobiological data collected in this study. Three main findings emerged. First, individuals with higher autistic traits showed reduced efficiency in extracting relevant visual information under increasing external noise. Second, they responded more slowly during global-incongruent processing, requiring attentional zoom-out and integration of spatially distributed local visual information. Third, in the secondary exploratory regression, visual-noise accuracy explained significant variance in the AQ Social Skills scores when entered alone, whereas global-incongruent Navon performance explained additional variance and was the only measure making a statistically significant unique contribution in the final model. These findings describe the visual-processing differences and exploratory associations with the self-reported AQ Social Skills within the selected sample.

### 5.1. Visual Signal Extraction Under External Noise

The present findings indicate that individuals with higher autistic traits showed reduced accuracy in extracting visual information under increasing levels of external noise. Importantly, this effect did not emerge under low-noise conditions, suggesting that perceptual differences become apparent primarily when the visual system is required to segregate behaviourally relevant information from noisy visual input. Rather than reflecting a generalised impairment in visual perception, these findings are consistent with the hypothesis that individuals in the selected high-AQ group may show greater difficulty in processing complex visual scenes characterised by high levels of perceptual interference [37]. This interpretation is consistent with previous psychophysical studies showing that visual performance in autism is strongly influenced by the characteristics of the surrounding visual environment (e.g., [19,22]). Bertone et al. [37] first demonstrated that individuals with ASD exhibit atypical sensitivity to second-order visual information despite preserved performance for simpler visual stimuli, suggesting that perceptual differences emerge primarily when visual processing becomes computationally demanding. More recently, Park et al. [39] proposed that perceptual performance in ASD can be understood within an external-noise framework, whereby behavioural differences become most evident when the visual system must efficiently filter irrelevant information while preserving the representation of the target stimulus. Within this perspective, our findings extend previous observations by showing that high-AQ scores in a selected non-clinical sample are associated with reduced efficiency in extracting coherent object information from noisy visual scenes. Although the present behavioural data do not directly assess neural mechanisms, reduced performance under external noise is consistent with the possibility that individual differences in early visual encoding may contribute to the extraction of behaviourally relevant information before higher-order object representations are constructed.

### 5.2. Global Visual Integration and Attentional Deployment

The second main finding showed that participants in the high-AQ group responded more slowly during the global-incongruent condition of the Navon task, whereas no group differences emerged under the local incongruent condition. The absence of a global processing advantage on incongruent trials in the high-AQ group, in contrast to the advantage observed in the low-AQ group, may indicate greater difficulty prioritizing the global configuration when conflicting local information must be suppressed. Because incongruent displays require the observer to overcome interference from local elements while integrating information across spatially distributed regions of the visual field, these findings suggest that the observed group difference was evident when global perceptual organisation competes with locally salient information.

This interpretation is broadly consistent with previous accounts proposing atypical global visual processing in ASD [9]. Weak Central Coherence theory originally suggested that individuals with ASD show a reduced tendency to integrate local information into coherent global representations [12,13,14]. However, subsequent evidence has indicated that global processing is not uniformly impaired but rather depends on task demands and stimulus characteristics, revealing a difference in the temporal pattern of the local–global balance, that is, slow global processing in individuals with ASD [9]. The present findings are compatible with this view, as group differences emerged specifically when participants were required to extract the global configuration while suppressing conflicting local information, rather than during global processing per se. One possible interpretation is that the increased response times observed under the global-incongruent condition reflect reduced efficiency in attentional mechanisms supporting the integration of spatially distributed visual information [21]. This interpretation is consistent with studies showing that global perception preferentially recruits the stimulus-driven reorienting system of the ventral attention network, particularly within the right hemisphere [25,26,46,47], and with the proposal that expanding the attentional focus (“attentional zoom-out”) constitutes a critical prerequisite for efficient global integration [18,27]. Importantly, the present behavioural data do not directly identify the neural mechanisms underlying this effect. Nevertheless, the observed behavioural pattern is compatible with the hypothesis that slower global integration may reflect less efficient deployment of attentional resources across complex visual scenes.

The present results are also compatible with recent neurobiological accounts proposing atypical magnocellular processing in ASD. In particular, Schelinski et al. [36] reported reduced magnocellular pathway functioning together with altered dorsal stream responses in adults with ASD, suggesting that early visual encoding may contribute to subsequent perceptual difficulties under challenging viewing conditions.

### 5.3. A Tentative Theoretical Framework of Visual Integration

The two tasks were selected to probe different aspects of visual processing, but the present data do not establish that they reflect distinct stages of a common mechanism. In the exploratory regression, visual-noise accuracy explained variance when entered alone, whereas global-incongruent Navon performance explained additional variance and was the only measure making a statistically significant unique contribution in the final model. These results, therefore, do not demonstrate independent contributions from both measures. Instead, they provide preliminary motivation for considering whether the two behavioural measures may be related to partially overlapping visual integration processes.

One possible theoretical context is provided by recent proposals that move beyond the traditional dichotomy between dorsal (“where”) and ventral (“what”) visual processing. Ayzenberg and Behrmann [32] proposed that dorsal computations contribute to constructing global structural descriptions of visual scenes, providing an intermediate representation that supports subsequent ventral object recognition. Rather than serving exclusively attentional or spatial functions, the dorsal stream may, therefore, play an active role in organizing spatially distributed visual information into coherent structural representations. The present behavioural findings are compatible with this framework, as both global visual integration and visual signal extraction under external noise may depend, at least in part, on the efficiency of these intermediate computations. However, the present data do not directly test this neural architecture and should, therefore, be interpreted as behavioural evidence consistent with, rather than demonstrating, this model.

Importantly, this framework may also provide a hypothesis for investigating possible links between object and social perception. Pitcher and Ungerleider [10] proposed that the perception of socially relevant information relies on a third visual pathway that partially overlaps with the ventral attentional network [25] while remaining functionally distinct from the classical ventral object-recognition pathway. Because both the ventral attention network and the social perception pathway receive substantial magnocellular input through area MT/V5 and are predominantly right-lateralised within temporoparietal cortex, efficient integration of spatially distributed visual information may constitute a computational prerequisite shared by both systems. Within this interpretation, behavioural differences in global integration and visual signal extraction could ultimately influence not only object perception but also the extraction of socially relevant visual cues [18,20].

Viewed from this perspective, several influential theories of atypical perception in autism may be understood as emphasizing different levels of the same neurocognitive architecture rather than representing mutually exclusive explanations. Weak Central Coherence primarily describes the behavioural consequences of reduced global integration [13,14], and Enhanced Perceptual Functioning emphasizes atypical weighting of local versus global information [15], whereas more recent neurobiological models have highlighted alterations in magnocellular processing [36], zoom-out attentional mechanisms [18,20], and dorsal structural representations [32]. Recent evidence further suggests that this architecture should also be considered from a temporal perspective. Prolonged neural encoding of visual representations in autism [48] indicates that atypical perception may depend not only on how visual information is processed but also on how long sensory representations remain active before being updated. This temporal dimension may interact with magnocellular, attentional, and dorsal-stream mechanisms to shape the efficiency of visual integration [49]. The present findings do not distinguish among these models or directly test their neural and temporal predictions. The framework presented here should, therefore, be regarded as a hypothesis-generating interpretation of the behavioural results rather than as evidence for successive computational stages linking visual processing to social functioning. The theoretical model illustrated in Figure 6 will require direct testing in larger, continuously sampled and clinically characterised populations using neural measures and direct assessments of social perception and functioning.

### 5.4. Limitations and Future Directions

Several limitations should be considered when interpreting the present findings. First, the small analytical samples provided limited sensitivity to small effects and reduced the precision of the estimated associations. In addition, the cross-sectional design precludes conclusions regarding the developmental or causal relationships between visual integration mechanisms and self-reported social-skills-related autistic traits. Longitudinal studies will be necessary to determine whether atypical visual integration precedes and contributes to individual differences in social abilities (e.g., [8,11]) or instead reflects downstream consequences of broader neurodevelopmental processes [41].

Second, participants were selected using an extreme-groups approach based on autistic traits (e.g., [27]). Although this strategy increases statistical sensitivity for detecting group differences, it excludes individuals with intermediate AQ scores and may produce less stable or inflated effect estimates. This issue is particularly relevant to the exploratory regression because the AQ Social Skills subscale is a component of the AQ total score used for participant selection. The regression, therefore, describes associations within this selected sample and should not be interpreted as an estimate across the full autism-trait continuum. Future studies should examine whether the present associations generalize across the full continuum of autistic traits using larger population-based samples.

A further limitation concerns the demographic composition of the sample, which consisted of young university students and was predominantly female. Given accumulating evidence that autistic traits and perceptual processing may differ between males and females, the generalizability of the present findings requires replication in more balanced samples [50]. Moreover, participants were drawn from a non-clinical population and did not have a confirmed ASD diagnosis. High AQ scores should not be interpreted as equivalent to an ASD diagnosis, and the present findings cannot be generalised directly to autistic individuals. In addition, the AQ Social Skills subscale is a self-report measure rather than a direct assessment of everyday social functioning. No clinician-rated, informant-reported, behavioural, or ecologically valid measures of social functioning were included. Although the published psychometric properties of the AQ and its Social Skills subscale are reported, their internal consistency was not estimated in the present sample; this represents an additional measurement limitation. Potential confounders, including general cognitive ability, basic processing speed, attentional or emotional symptoms, and medication use, were not systematically assessed.

Finally, the present study relied exclusively on behavioural measures. Consequently, although our interpretation was informed by contemporary models of magnocellular processing, attentional networks, and dorsal structural representations [18,27,32,36], the involvement of these neural mechanisms cannot be directly inferred from the current data. Combining psychophysical paradigms with neuroimaging, electrophysiology, or non-invasive brain stimulation will be essential to test the proposed computational framework more directly.

Future studies should also investigate whether the visual integration mechanisms identified here are amenable to targeted cognitive training. Evidence from other neurodevelopmental conditions frequently co-occurring with ASD suggests that the local–global balance may be modifiable. In children with developmental dyslexia, approximately 12 h of action video-game training reduced local interference during global processing and increased the influence of global information during local processing [41]. Moreover, even a single hour of engaging video-game play improved performance selectively in the global-incongruent condition in children with developmental dyslexia and developmental coordination disorder, although this short-term effect may reflect both action-game characteristics and the positive emotional state elicited by play [51]. These findings raise the possibility that interventions enhancing broad visuospatial attention and the integration of distributed information could also modulate the perceptual mechanisms. This possibility should be investigated using direct measures of social functioning in individuals with ASD and in samples characterised by co-occurring neurodevelopmental conditions.

Moreover, the two behavioural tasks were selected to probe complementary aspects of visual integration rather than to isolate specific neural pathways. Future studies combining psychophysical paradigms with pathway-selective stimuli and multimodal neuroimaging will be important to determine how individual stages of the proposed framework map onto their underlying neural substrates.

## 6. Conclusions

The present study identified differences in the following two aspects of visual processing between young adults selected for high and low AQ scores: visual signal extraction under external noise and global-incongruent processing. In the secondary exploratory regression within this selected sample, visual-noise accuracy explained significant variance when entered alone, whereas global-incongruent Navon performance explained additional variance and was the only measure making a statistically significant unique contribution in the final model. These findings suggest that the two visual-processing measures may provide complementary information about AQ Social Skills scores within the selected sample, but they do not establish independent contributions from both measures, associations across the broader autism-trait continuum, or relationships with everyday social functioning.

The framework presented in Figure 6 offers a hypothesis-generating interpretation linking early visual encoding, attentional integration, and socially relevant perception. The present behavioural data neither directly test the proposed magnocellular, dorsal-stream, or neural mechanisms nor establish a common computational substrate. Larger continuously sampled and clinically characterised studies, combining behavioural tasks with direct measures of social functioning and neural methods, are required to evaluate this framework.

## Data Availability

Unrestricted public deposition of the individual-level data is not permitted because the informed-consent procedure and ethical approval did not include public data sharing. A de-identified analysis dataset, SPSS version 32.0 analysis syntax, variable codebook, and the documentation of the preprocessing and statistical procedures may be made available to qualified researchers through controlled access following a methodologically justifiable submission request to the corresponding author and completion of an appropriate data-use agreement. Data will be shared only for research purposes and in accordance with the applicable ethical and data-protection requirements.

## Abbreviations

The following abbreviations are used in this manuscript:

ANOVA	Analysis of Variance
AQ	Autism-Spectrum Quotient
ASD	Autism Spectrum Disorder
MRI	Magnetic Resonance Imaging
MT/V5	Middle Temporal Visual Area, also known as Visual Area V5
RDoC	Research Domain Criteria
SD	Standard Deviation
SEM	Standard Error of the Mean
